# Triggering of Programmed Erythrocyte Death by Alantolactone

**DOI:** 10.3390/toxins6123596

**Published:** 2014-12-22

**Authors:** Kousi Alzoubi, Salvatrice Calabrò, Jasmin Egler, Caterina Faggio, Florian Lang

**Affiliations:** 1 Department of Physiology, University of Tübingen, Gmelinstr. 5, 72076 Tuebingen, Germany; E-Mails: kossai.z@gmail.com (K.A.); salva24@inwind.it (S.C.); jasmin.egler@t-online.de (J.E.); 2 Department of Biological and Environmental Sciences, University of Messina, Viale Ferdinando Stagno d’Alcontres 31, 98166 S. Agata-Messina, Italy; E-Mail: cfaggio@unime.it

**Keywords:** phosphatidylserine, alantolactone, ceramide, oxidative stress, cell volume, eryptosis

## Abstract

The sesquiterpene alantolactone counteracts malignancy, an effect at least in part due to stimulation of suicidal death or apoptosis of tumor cells. Signaling of alantolactone induced apoptosis involves altered gene expression and mitochondrial depolarization. Erythrocytes lack mitochondria and nuclei but may enter suicidal death or eryptosis, which is characterized by cell shrinkage and cell membrane scrambling with phosphatidylserine exposure at the erythrocyte surface. Cellular mechanisms involved in triggering of eryptosis include increase of cytosolic Ca^2+^-activity ([Ca^2+^]*_i_*) and oxidative stress. The present study explored, whether alantolactone stimulates eryptosis. To this end, erythrocyte volume was estimated from forward scatter, phosphatidylserine-exposure at the erythrocyte surface from FITC-annexin-V-binding, [Ca^2+^]*_i_* from Fluo3-fluorescence, ceramide abundance from binding of fluorescent antibodies, and oxidative stress from 2',7'-dichlorodihydrofluorescein-diacetate (DCFDA) fluorescence. As a result, a 48 h exposure of human erythrocytes to alantolactone (≥20 μM) significantly decreased erythrocyte forward scatter and increased the percentage of annexin-V-binding cells. Alantolactone significantly increased Fluo3 fluorescence (60 μM), ceramide abundance (60 μM) and DCFDA fluorescence (≥40 μM). The effect of alantolactone (60 μM) on annexin-V-binding was not significantly modified by removal of extracellular Ca^2+^. In conclusion, alantolactone stimulates suicidal erythrocyte death or eryptosis, an effect paralleled by increase of [Ca^2+^]*_i_*, ceramide abundance and oxidative stress.

## 1. Introduction

Alantolactone, a sesquiterpene isolated from several medicinal plants [1], counteracts inflammation, infection and malignancy [1]. Its anticancer efficacy is attributed to its ability to induce apoptosis of tumor cells [1]. Alantolactone triggers apoptosis of a variety of cells [2,3,4,5,6,7,8,9,10,11]. Cellular mechanisms involved in the stimulation of apoptosis by alantolactone include disruption of mitochondrial membrane potential [3,6,8,11], induction of oxidative stress [3,4,8,11], interference with gene expression [3,4,6,7,9,10], increased Bax/Bcl-2 ratio [3,7,10] and activation of caspases [3,6,7,8,10].

Erythrocytes lack mitochondria and nuclei and are thus resistant to triggers of suicidal death effective by mitochondrial depolarization or altered gene expression [12]. Nevertheless, erythrocytes may undergo apoptosis-like suicidal death or eryptosis, which is characterized by cell shrinkage and break down of cell membrane phospholipid asymmetry with translocation of phosphatidylserine to the cell surface [12]. Stimulators of eryptosis include increase of cytosolic Ca^2+^ concentration ([Ca^2+^]_i_), which activates Ca^2+^-sensitive K^+^ channels with subsequent K^+^ exit, hyperpolarization, Cl^−^ exit and thus cell shrinkage due to cellular loss of KCl with water [13]. Increase of [Ca^2+^]_i_ further leads to translocation of phosphatidylserine to the erythrocyte surface [12]. Cellular mechanisms triggering eryptosis further include ceramide [14], oxidative stress [15], activated caspases [16,17,18,19,20] decreased activities of AMP activated kinase AMPK [21], cGMP-dependent protein kinase [17], PAK2 kinase [22], sorafenib sensitive kinases [23] and sunifinib sensitive kinases [24], or excessive activities of casein kinase 1α [25,26], Janus-activated kinase JAK3 [27], protein kinase C [28], and p38 kinase [29].

Eryptosis has been extensively studied in both human and murine erythrocytes [12]. The involved mechanisms are similar but not necessarily identical [12]. Eryptosis is stimulated by a wide variety of chemicals [14,23,24,30,31,32,33,34,35,36,37,38,39,40,41,42,43,44,45,46,47,48,49,50,51,52,53,54,55,56,57,58,59,60,61,62,63,64,65] and excessive eryptosis is observed in several clinical conditions, including sepsis, fever, malaria, sickle cell disease, thalassemia, Wilson’s disease, iron deficiency, hepatic failure, malignancy, metabolic syndrome, diabetes, dehydration, renal insufficiency, hemolytic uremic syndrome, hyperphosphatemia and phosphate depletion [12,66,67].

The present study explored, whether eryptosis is stimulated by alantolactone. To this end, human erythrocytes drawn from healthy volunteers were exposed to alantolactone and cell volume, phosphatidylserine abundance at the cell surface, [Ca^2+^]_i_ and reactive oxygen species (ROS) determined.

## 2. Results and Discussion

In order to test whether the sesquiterpene alantolactone triggers eryptosis, the suicidal erythrocyte death, human erythrocytes were exposed for 48 h to Ringer solution without or with alantolactone (10–60 μM) and cell volume as well as phosphatidylserine translocation to the erythrocyte surface were determined.

**Figure 1:**
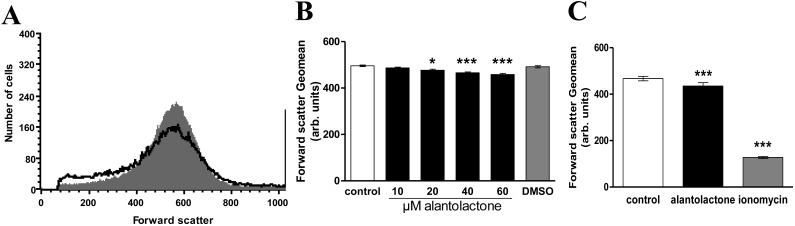
Effect of alantolactone on erythrocyte forward scatter. (**A**) Original histogram of forward scatter of erythrocytes following exposure for 48 h to Ringer solution without (grey area) and with (black line) presence of 60 μM alantolactone; (**B**) Arithmetic means ± SEM (*n* = 15) of the normalized erythrocyte forward scatter (FSC) following incubation for 48 h to Ringer solution without (white bar) or with (black bars) alantolactone (10–60 μM). For comparison, the effect of 1 μL DMSO/mL Ringer is shown (grey bar). * (*p* < 0.05), *** (*p* < 0.001) indicates significant difference from the absence of alantolactone (ANOVA); (**C**) Arithmetic means ± SEM (*n* = 4) of the normalized erythrocyte forward scatter (FSC) following incubation for 48 h to Ringer solution without alantolactone (white bar), or following 48 h treatment with 60 μM alantolactone (black bar) or following 1 h treatment with 1 μM ionomycin (grey bar). *** (*p* < 0.001) indicates significant difference from the absence of treatment (ANOVA).

Forward scatter was determined utilizing flow cytometry in order to estimate alterations of cell volume. As shown in Figure 1, a 48 h exposure to alantolactone-containing Ringer was followed by a decrease of forward scatter, an effect reaching statistical significance at 20 μM alantolactone concentration.

**Figure 2:**
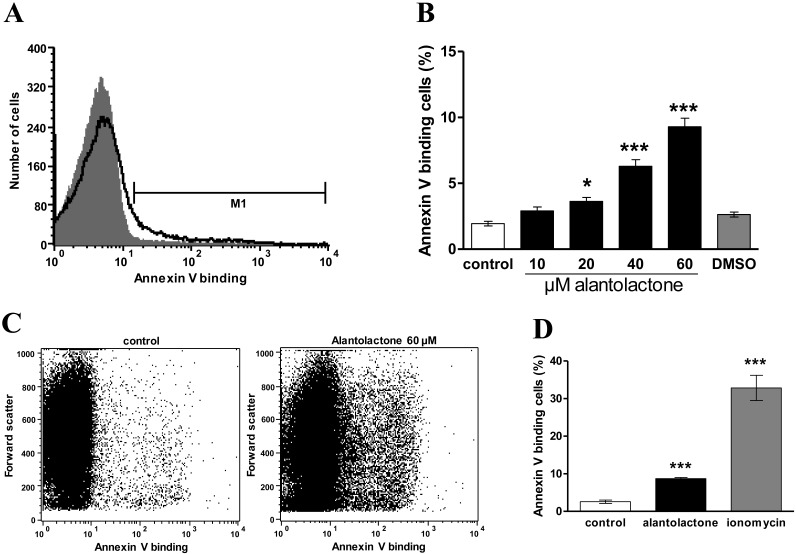

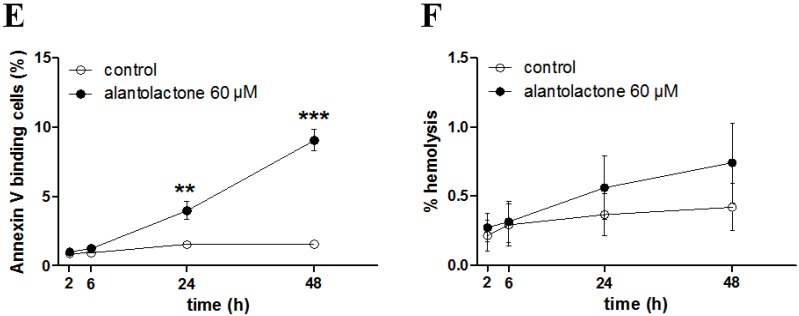
Effect of alantolactone on phosphatidylserine exposure. (**A**) Original histogram of annexin-V-binding erythrocytes following exposure for 48 h to Ringer solution without (grey area) and with (black line) presence of 60 µM alantolactone; (**B**) Arithmetic means ± SEM (*n* = 15) of erythrocyte annexin-V-binding following incubation for 48 h to Ringer solution without (white bar) or with (black bars) presence of alantolactone (10–60 µM). For comparison, the effect of 1 µL DMSO/mL Ringer is shown (grey bar). * (*p* < 0.05), *** (*p* < 0.001) indicate significant difference from the absence of alantolactone (ANOVA); (**C**) Original dot plots of forward scatter vs annexin-V-FITC binding of erythrocytes following exposure for 48 h to Ringer solution without (left) and with (right) presence of 60 µM alantolactone; (**D**) Arithmetic means ± SEM (*n* = 4) of the erythrocyte annexin V binding following incubation for 48 h to Ringer solution without treatment (white bar), following a 48 h treatment with 60 µM alantolactone (black bar) or following a 1 h treatment with 1 µM ionomycin (grey bar). *** (*p* < 0.001) indicates significant difference from the absence of treatment (ANOVA); (**E**) Arithmetic means ± SEM (*n* = 4) of the percentage annexin V binding erythrocytes as a function of exposure time to Ringer without (open circles) or with (closed circles) alantolactone (60 µM). ** (*p* < 0.01), *** (*p* < 0.001) indicate significant differences from absence of alantolactone (ANOVA); (**F**) Arithmetic means ± SEM (*n* = 4) of percentage of hemolysis as a function of exposure time to Ringer without (open circles) or with (closed circles) alantolactone (60 µM).

Phosphatidylserine translocation to the erythrocyte surface was quantified from binding of FITC-labelled annexin-V as determined in flow cytometry. As shown in Figure 2, a 48 h exposure to alantolactone was followed by an increase of the percentage of erythrocytes binding FITC-labelled annexin-V, an effect reaching statistical significance at 20 μM alantolactone concentration.

Hemolysis was estimated by determination of hemoglobin in the supernatant. As shown in Figure 2F, alantolactone tended to slightly increase the percentage of hemolysed erythrocytes, an effect, however, not reaching statistical significance.

**Figure 3:**
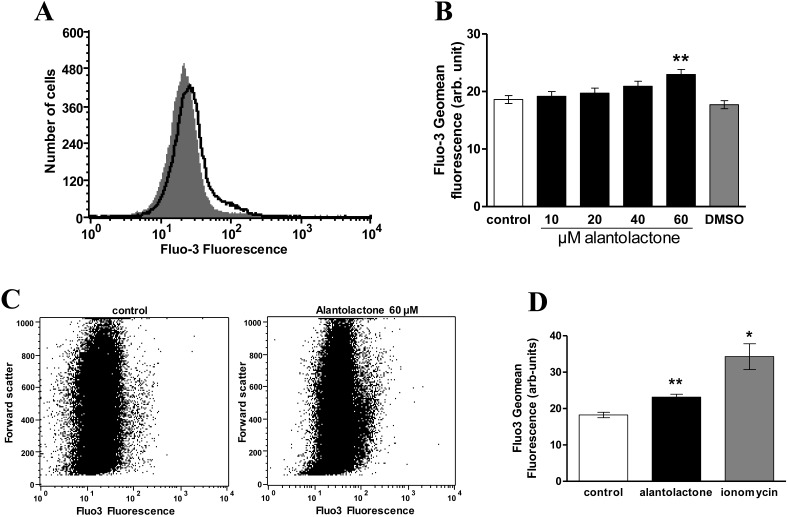
Effect of alantolactone on Fluo3 fluorescence. (**A**) Original histogram of Fluo3-fluorescence following exposure for 48 h to Ringer solution without (grey area) and with (black line) presence of 60 µM alantolactone; (**B**) Arithmetic means ± SEM (*n* = 15) of erythrocyte Fluo3-fluorescence following incubation for 48 h to Ringer solution without (white bar) or with (black bars) presence of alantolactone (10–60 µM). For comparison, the effect of 1 µL DMSO/mL Ringer is shown (grey bar). ** (*p* < 0.01) indicate significant difference from the absence of alantolactone (ANOVA); (**C**) Original dot plots of forward scatter vs fluo3 fluorescence of erythrocytes following exposure for 48 h to Ringer solution without (**left**) and with (**right**) presence of 60 µM alantolactone; (**D**) Arithmetic means ± SEM (*n* = 4) of the erythrocyte Fluo-3 fluorescence following incubation for 48 h to Ringer solution without treatment (white bar), following a 48 h treatment with 60 µM alantolactone (black bars) or following 1 h treatment with 1 µM ionomycin (grey bar). * (*p* < 0.05), ** (*p* < 0.01) indicates significant difference from the absence of alantolactone (ANOVA).

Both, cell shrinkage and phosphatidylserine translocation to the cell surface could have resulted from an increase of cytosolic Ca^2+^ activity ([Ca^2+^]_i_). Thus, additional experiments explored the effect of alantolactone on [Ca^2+^]_i_. Following a 48 h incubation in Ringer solution without or with alantolactone (10–60 µM), the erythrocytes were loaded with Fluo3-AM and the Fluo3 fluorescence determined by flow cytometry. As shown in Figure 3, exposure of the erythrocytes to alantolactone was followed by an increase of Fluo3 fluorescence, an effect reaching statistical significance at 60 µM alantolactone concentration.

**Figure 4:**
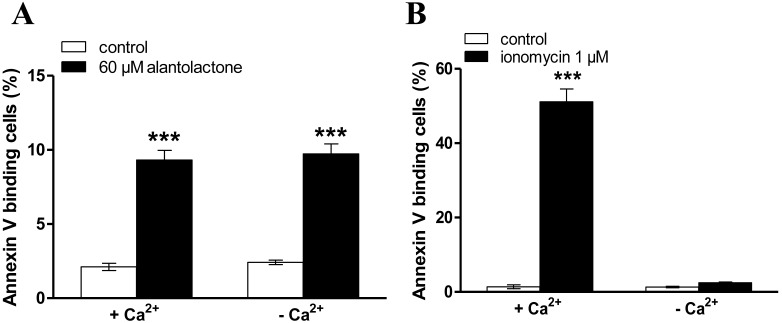
Effect of Ca^2+^ withdrawal on alantolactone- or ionomycin-induced phosphatidylserine exposure. (**A**) Arithmetic means ± SEM (*n* = 10) of annexin-V-binding erythrocytes after a 48 h treatment with Ringer solution without (white bar) or with (black bars) 60 µM alantolactone in the presence (left bars, +Ca^2+^) and absence (right bars, −Ca^2+^) of calcium. *** (*p* < 0.001) indicates significant difference from the respective values in the absence of alantolactone (ANOVA); (**B**) Arithmetic means ± SEM (*n* = 4) of annexin-V-binding erythrocytes after a 1 h treatment with Ringer solution without (white bar) or with (black bars) 1 µM ionomycin in the presence (left bars, +Ca^2+^) and absence (right bars, −Ca^2+^) of calcium. *** (*p* < 0.001) indicates significant difference from the respective values in the absence of ionomycin (ANOVA).

In order to test whether the alantolactone induced cell membrane scrambling required entry of extracellular Ca^2+^, erythrocytes were exposed for 48 h to 60 µM alantolactone in the presence or nominal absence of extracellular Ca^2+^. As illustrated in Figure 4A, the effect of alantolactone on annexin-V-binding was not significantly modified by removal of extracellular Ca^2+^. Thus, the effect of alantolactone on annexin-V-binding did not depend on Ca^2+^ entry. To ascertain that the high calcium content (5 mM) in the staining solution did not affect the results, the effect ionomycin was studied using the same protocol. As illustrated in Figure 4B, the effect of ionomycin on annexin-V-binding was completely abrogated by removal of extracellular Ca^2+^.

**Figure 5:**
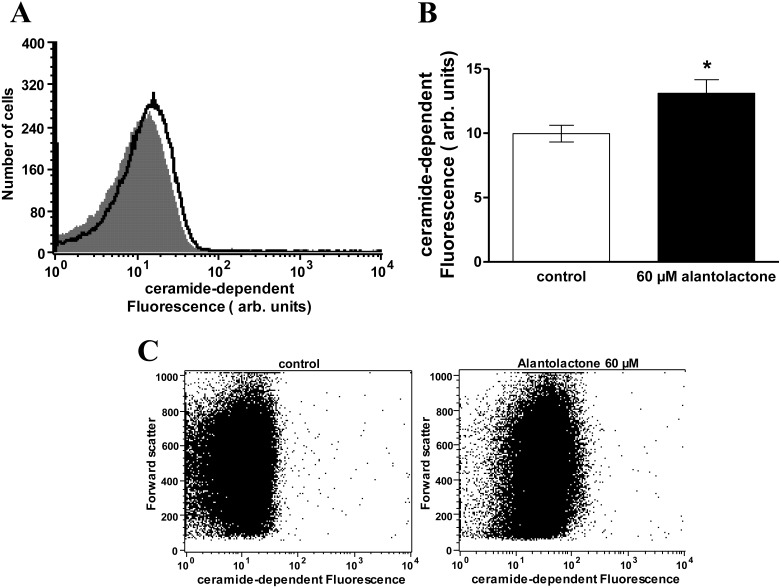
Effect of alantolactone on ceramide formation. (**A**) Original histogram of ceramide surface abundance of erythrocytes following exposure for 48 h to Ringer solution without (grey shadow) and with (black line) presence of 60 µM alantolactone; (**B**) Arithmetic means ± SEM (*n* = 10) of ceramide abundance after a 48 h incubation in Ringer solution without (white bar) or with 60 µM alantolactone (black bar). * (*p* < 0.05) indicates significant difference from the absence of alantolactone (*t* test); (**C**) Original dot plots of forward scatter vs ceramide dependent fluorescence of erythrocytes following exposure for 48 h to Ringer solution without (**left**) and with (**right**) presence of 60 µM alantolactone.

Mechanisms stimulating eryptosis without increase of [Ca^2+^]*_i_* include ceramide. Thus, additional experiments were performed in order to quantify the effect of alantolactone on the ceramide abundance at the erythrocyte surface. To this end the ceramide abundance was determined utilizing a fluorescent anti-ceramide antibody. As shown in Figure 5, a 48 h exposure of erythrocytes to 60 µM alantolactone significantly increased the abundance of ceramide at the erythrocyte surface.

**Figure 6:**
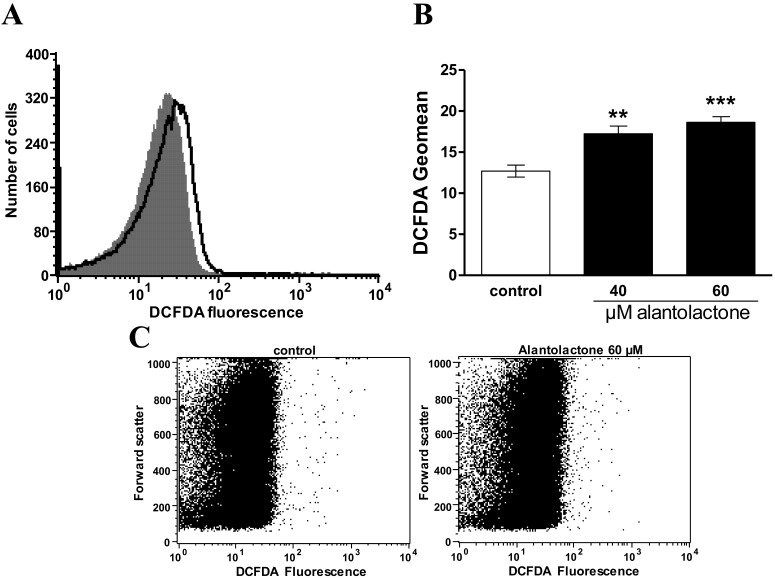
Effect of alantolactone on DCFDA fluorescence. (**A**) Original histogram of 2',7'-dichlorodihydrofluorescein diacetate (DCFDA) fluorescence following exposure for 48 h to Ringer solution without (grey area) and with (black line) presence of 60 µM alantolactone; (**B**) Arithmetic means ± SEM (*n* = 10) of erythrocyte DCFDA-fluorescence following incubation for 48 h to Ringer solution without (white bar) or with (black bars) presence of alantolactone (40–60 µM). ** (*p* < 0.01), *** (*p* < 0.001) indicates significant difference from the absence of alantolactone (ANOVA); (**C**) Original dot plots of forward scatter *vs.* DCFDA fluorescence of erythrocytes following exposure for 48 h to Ringer solution without (**left**) and with (**right**) presence of 60 µM alantolactone.

Eryptosis is further triggered by oxidative stress. Thus, DCFDA fluorescence was determined to estimate reactive oxygen species (ROS). As illustrated in Figure 6, a 48 h exposure to alantolactone (40 or 60 µM) was followed by a significant increase of DCFDA fluorescence pointing to induction of oxidative stress.

Additional experiments addressed the effect of alantolactone on flippase and scramblase activity. To this end, erythrocytes were exposed for 48 h to alantolactone (60 µM) and the uptake of NBD-PS-(1-palmitoyl-2-[6-[(7-nitro-2-1,3-benzoxadiazol-4-yl)amino]-hexanoyl]-*sn*-glycero-3-phos-phoserine) determined as a measure of flippase activity and the uptake of NBD-PC (1-Oleoyl-2-[12-[(7-nitro-2-1,3-benzoxadiazol-4-yl)amino]dodecanoyl]-sn-Glycero-3-Phosphocholine) determined as a measure of scramblase activity. Within 60 min the NBD-PS uptake was significantly lower in alantolactone treated erythrocytes (7.9% ± 0.3%, *n* = 4) than in untreated erythrocytes (22.8% ± 1.1%, *n* = 4), whereas the NBD-PC uptake was significantly higher in alantolactone treated erythrocytes (73.9% ± 3.1%, *n* = 4) than in untreated erythrocytes (52.8% ± 1.2%, *n* = 4).

The present study reveals that alantolactone triggers eryptosis, the suicidal death of erythrocytes, *i.e.*, of cells without mitochondria and without nuclei and thus unable to undergo mitochondrial triggering of cell death or modifying the expression of proteins involved in the machinery leading to cell death.

Apparently higher concentrations of alantolactone are required to appreciably increase [Ca^2+^]*_i_* than the alantolactone concentrations required for stimulation of phosphatidylserine translocation or induction of cell shrinkage. Moreover, the removal of extracellular Ca^2+^ did not appreciably modify alantolactone induced cell membrane scrambling. Thus, entry of extracellular Ca^2+^ cannot account for the stimulation of eryptosis by alantolactone. Instead, stimulation of eryptosis by alantolactone presumably involves ceramide formation and induction of oxidative stress. NBD-PS and NBD-PC uptake studies point to the modification of flippase and scramblase activities.

The cell shrinkage, which is presumably in part due to due to activation of Ca^2+^ sensitive K^+^ channels and subsequent cellular loss of KCl and water [13], is only mild and much less pronounced than the cell shrinkage induced by the Ca^2+^ ionophore ionomycin. Close inspection of the histogram in Figure 1 reveals that a small subpopulation of erythrocytes even rather swells. Possibly, alantolactone stimulated Na^+^ entry, which may in some cells override K^+^ exit. In contrast to its strong effect on eryptosis, alantolactone treatment tended to only slightly increase hemolysis, an effect not reaching statistical significance (Figure 2).

The cell shrinkage serves to counteract cell swelling and the phosphatidylserine exposure at the cell surface is an “eat me” signal leading to phagocytosis of eryptotic cells. The triggering of eryptosis thus counteracts hemolysis of defective erythrocytes. The hemolysis would otherwise be followed by release of hemoglobin, which may be filtered in renal glomeruli and subsequently precipitate in the acidic lumen of renal tubules [68]. The removal of eryptotic erythrocytes is an important host defence mechanism during infection with *Plasmodia* [69]. The intraerythrocytic parasite activates several ion channels in the host cell membrane including the Ca^2+^-permeable erythrocyte cation channels [70,71]. The subsequent Ca^2+^ entry triggers eryptosis with subsequent clearance of the infected erythrocytes from circulating blood [69,72]. Accordingly, genetic disorders sensitizing erythrocytes to eryptosis, such as sickle-cell trait, beta-thalassemia-trait, homozygous Hb-C and G6PD-deficiency [12,73,74,75], lead to accelerated eryptosis of infected erythrocytes thus counteracting parasitemia and a severe course of the disease [69]. Similarly, some clinical conditions fostering eryptosis, such as iron deficiency [72], and several eryptosis stimulating xenobiotics, such as lead [76], chlorpromazine [77] or NO synthase inhibitors [78] have been shown to favourably influence the clinical course of malaria. It remains to be tested, whether alantolactone influences the clinical course of malaria.

The *in vivo* clearance of eryptotic erythrocytes from circulating blood [12,79,80,81,82] may result in anemia, as soon as the rate of eryptosis with subsequent clearance from circulating blood exceeds the formation of new erythrocytes [12]. Moreover, phosphatidylserine exposing erythrocytes adhere to endothelial CXCL16/SR-PSO [83], stimulate blood clotting and thrombosis [84,85,86] and thus interfere with microcirculation [83,84,87,88,89,90].

The alantolactone concentrations required for stimulation of eryptosis were similar to those effective in cancer cells [2,3,4,5,6,7,8,9,10,11]. In theory, enhanced eryptosis may thus limit the use of alantolactone in the treatment of tumors. It must be kept in mind that eryptosis is enhanced in malignancy [82], a complication presumably compounded by therapeutic use of eryptosis inducing substances.

## 3. Experimental Section

### 3.1. Erythrocytes, Solutions and Chemicals

Fresh Li-Heparin-anticoagulated blood samples were kindly provided by the blood bank of the University of Tübingen. The study is approved by the ethics committee of the University of Tübingen (184/2003 V). The blood was centrifuged at 120 rcf for 20 min at room temperature and the platelets and leukocytes-containing supernatant was disposed. Erythrocytes were incubated *in vitro* at a hematocrit of 0.4% in Ringer solution containing (in mM) 125 NaCl, 5 KCl, 1 MgSO4, 32 *N*-2-hydroxyethylpiperazine-*N*-2-ethanesulfonic acid (HEPES), 5 glucose, 1 CaCl_2_; pH 7.4 at 37 °C for 48 h. Where indicated, erythrocytes were exposed to alantolactone (Sigma Aldrich, Schnelldorf, Germany) at the indicated concentrations, solved in 1 µL/mL DMSO. For comparison, the effect of 1 µL DMSO/mL Ringer was tested.

### 3.2. Analysis of Annexin-V-Binding and Forward Scatter

After incubation under the respective experimental condition, 150 µL cell suspension was washed in Ringer solution containing 5 mM CaCl_2_ and then stained with Annexin-V-FITC (1:200 dilution; ImmunoTools, Friesoythe, Germany) in this solution at 37 °C for 20 min under protection from light. In the following, the forward scatter (FSC) of the cells was determined, and annexin-V fluorescence intensity was measured with an excitation wavelength of 488 nm and an emission wavelength of 530 nm on a FACS Calibur (BD, Heidelberg, Germany). The incubation with Annexin-V-FITC required presence of 5 mM CaCl_2_ even in experiments on the effect of a 48 h incubation with alantolactone in the absence of Ca^2+^. In order to test whether the short incubation with 5 mM Ca^2+^ could have biased the results, experiments were performed in erythrocytes treated with Ca^2+^ ionophore ionomycin. As indicated in Figure 4, a 20 min exposure to extracellular Ca^2+^ in the presence of Ca^2+^ ionophore ionomycin (1 µM) was not sufficient to trigger significant annexin-V binding.

### 3.3. Measurement of Intracellular Ca^2+^

After incubation erythrocytes were washed in Ringer solution and then loaded with Fluo-3/AM (Biotium, Hayward, CA, USA) in Ringer solution containing 5 mM CaCl_2_ and 5 µM Fluo-3/AM. The cells were incubated at 37 °C for 30 min and washed twice in Ringer solution containing 5 mM CaCl_2_. The Fluo-3/AM-loaded erythrocytes were resuspended in 200 µL Ringer. Then, Ca^2+^-dependent fluorescence intensity was measured with an excitation wavelength of 488 nm and an emission wavelength of 530 nm on a FACS Calibur.

### 3.4. Determination of Ceramide Formation

To determine ceramide abundance, a monoclonal antibody-based assay was used. After incubation, cells were stained for 1 h at 37 °C with 1 μg/mL anti-ceramide antibody (clone MID 15B4; Alexis, Grünberg, Germany) in phosphate-buffered saline (PBS) containing 0.1% bovine serum albumin (BSA) at a dilution of 1:10. After two washing steps with PBS-BSA, cells were stained for 30 min with polyclonal fluorescein- isothiocyanate (FITC)-conjugated goat anti-mouse IgG and IgM specific antibody (Pharmingen, Hamburg, Germany) diluted 1:50 in PBS-BSA. Unbound secondary antibody was removed by repeated washing with PBS-BSA. Samples were then analyzed by flow cytometric analysis at an excitation wavelength of 488 nm and an emission wavelength of 530 nm.

### 3.5. Determination of Reactive Oxygen Species (ROS)

ROS production was determined utilizing 2',7'-dichlorodihydrofluorescein diacetate (DCFDA). Briefly, the cells were suspended in FACS buffer and the fluorescence was analysed with flow cytometry (FACS-Calibur from Becton Dickinson; Heidelberg, Germany). DCFDA fluorescence intensity was measured in FL-1 with an excitation wavelength of 488 nm and an emission wavelength of 530 nm.

### 3.6. Measurement of Phospholipid Translocation

Phospholipid translocation was measured according to methods describes previously [91]. Cells were incubated with 60 µM alantolactone for 48 h and then loaded with 2 µM of NBD-PS 1-palmitoyl-2-[6-[(7-nitro-2-1,3-benzoxadiazol-4-yl)amino]hexanoyl]-*sn*-glycero-3-phosphoserine as a measure of flippase activity or NBD-PC 1-Oleoyl-2-[12-[(7-nitro-2-1,3-benzoxadiazol-4-yl)amino]dodecanoyl]-sn-Glycero-3-Phosphocholine as a measure of scramblase activity. Both are from Avanti Polar Lipids (Alabaster, AL, USA). After 60 min aliquots were obtained and resuspended in ice cold PBS for 10 min in the presence or absence of 1% BSA. The amount of translocated probe was determined by dividing the mean fluorescence intensity of the sample after BSA extraction (internalized probe) by that in absence of BSA (total probe). Analysis was done using FACS-Calibur from Becton Dickinson; Heidelberg, Germany.

### 3.7. Statistics

Data are expressed as arithmetic means ± SEM. As indicated in the figure legends, statistical analysis was made using ANOVA with Tukey’s test as post-test and *t* test as appropriate. *n* denotes the number of different erythrocyte specimens studied. Since different erythrocyte specimens used in distinct experiments are differently susceptible to triggers of eryptosis, the same erythrocyte specimens have been used for control and experimental conditions.

## 4. Conclusions

Exposure of human erythrocytes to alantolactone is followed by stimulation of eryptosis, characterized by erythrocyte shrinkage and phosphatidylserine translocation to the erythrocyte surface. Signaling involved includes increase of [Ca^2+^]*_i_*, ceramide formation and induction of oxidative stress.
